# Epithelial–mesenchymal plasticity in cancer: signaling pathways and therapeutic targets

**DOI:** 10.1002/mco2.659

**Published:** 2024-08-01

**Authors:** Xiangpeng Wang, Xiaoxia Xue, Mingshi Pang, Liuchunyang Yu, Jinxiu Qian, Xiaoyu Li, Meng Tian, Aiping Lyu, Cheng Lu, Yuanyan Liu

**Affiliations:** ^1^ School of Materia Medica Beijing University of Chinese Medicine Beijing China; ^2^ School of Chinese Medicine Hong Kong Baptist University Kowloon Hong Kong China; ^3^ Institute of Basic Research in Clinical Medicine China Academy of Chinese Medical Sciences Beijing China

**Keywords:** cancer, cell plasticity, deterioration, EMT, signaling pathway

## Abstract

Currently, cancer is still a leading cause of human death globally. Tumor deterioration comprises multiple events including metastasis, therapeutic resistance and immune evasion, all of which are tightly related to the phenotypic plasticity especially epithelial–mesenchymal plasticity (EMP). Tumor cells with EMP are manifest in three states as epithelial–mesenchymal transition (EMT), partial EMT, and mesenchymal–epithelial transition, which orchestrate the phenotypic switch and heterogeneity of tumor cells via transcriptional regulation and a series of signaling pathways, including transforming growth factor‐β, Wnt/β‐catenin, and Notch. However, due to the complicated nature of EMP, the diverse process of EMP is still not fully understood. In this review, we systematically conclude the biological background, regulating mechanisms of EMP as well as the role of EMP in therapy response. We also summarize a range of small molecule inhibitors, immune‐related therapeutic approaches, and combination therapies that have been developed to target EMP for the outstanding role of EMP‐driven tumor deterioration. Additionally, we explore the potential technique for EMP‐based tumor mechanistic investigation and therapeutic research, which may burst vigorous prospects. Overall, we elucidate the multifaceted aspects of EMP in tumor progression and suggest a promising direction of cancer treatment based on targeting EMP.

## Graphical abstract

Epithelial–mesenchymal plasticity is a crucial mechanism that drive tumor progression, including metastasis, therapeutic resistance, and immune evasion. In this review, we summarized the complicated regulation of EMP and therapeutic strategies targeting EMP for cancer treatment.

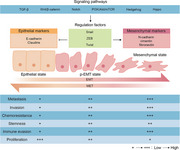

## 1 INTRODUCTION

Phenotypic plasticity, the ability that tumor cells gain various malignant phenotypes to better survive in the dynamically varying environment, is thought as the critical hallmark of tumor deterioration.^1^ Epithelial–mesenchymal plasticity (EMP) is one of the classically well‐studied manifestations of phenotypic plasticity during tumor progression.^2^ EMP represents the reversible switch of tumor cells from epithelial phenotype to partially or fully mesenchymal phenotype, including epithelial–mesenchymal transition (EMT), partial EMT (p‐EMT), and mesenchymal–epithelial transition (MET), which are observed during the process of tumor progression.^3^

EMP is regulated by various heterotypic signals originating from the dynamic tumor microenvironment, driving tumor cells to switch their phenotypes.^4^ The signals form the microenvironment for example transforming growth factor (TGF)‐β, epigenetically orchestrate the phenotypic switch of tumor cells via modulating the expression of a range of transcription factors (TFs), including Snail, ZEB, and Twist, which impact on the expression of epithelial genes or mesenchymal genes.^5^ With the changes in cellular phenotypes, tumor cells acquire different malignant features to adapt to the stress conditions. During tumor progression, tumor cells with EMP will be influenced by dynamic and stressful microenvironmental factors, such as the primary site with hypoxia, inflammation, remodeled extracellular matrix (ECM), and various stromal cells, the vascular environment with immune surveillance and the premetastatic site with extracellular vesicles (EVs), all of which may impact the EMP states of tumor cells via regulating EMP‐related signaling pathways. Furthermore, to maintain EMP and survival under stress, the stress conditions in environment will also lead to the activation of intracellular mechanisms involved in EMP, including metabolic reprogramming, domesticated autophagy, and anoikis. Fully understanding the mechanisms of EMP and the EMP‐driven tumor deterioration is an active area of research.

Currently, cancer is still the leading cause of human deaths worldwide.^6^ The high EMP of cancer cells has been widely observed to be tightly correlated with the poor prognosis of cancer, which can be attributed to EMP‐induced metastasis and therapeutic resistance.^7^, ^8^ Metastasis is a hallmark of cancer and is tightly associated with the different processes of EMP. EMT can promote the motility of cancer cells, enabling cancer cells to gain the ability to detach from their primary site and subsequently migrate to distant tissues, and p‐EMT results in collective cancer cell migration.^9^, ^10^ While MET is crucial for the colonization in the distant tissues, eventually leading to metastasis.^11^ Simultaneously, during EMT, the epithelial cells that transform into mesenchymal cells become resistant to chemotherapy, targeted therapies as well as immunotherapy, contributing to the treatment failure of cancer.^12^, ^13^ Besides, EMP is found to be implicated in the tumor heterogeneity and pathogenesis of some cancers.^10^, ^14^ Therefore, understanding the mechanisms, regulation and functions of EMP is of great significance for the cancer treatment.

In this review, we begin by introducing the processes of EMT and MET in cancer biology as well as their roles in phenotype switching and tumor heterogeneity. Given the crucial roles of transcriptional programs in determining cell identity, we then summarize the transcriptional regulation of EMP, including TFs and epigenetic factors regulating the expression of EMP‐related genes. Our discussion will further focus on the signaling pathways that modulate EMP and various intracellular and extracellular molecular mechanisms in the dynamic microenvironment that have impacts on EMP to provide a comprehensive overview of the EMP regulatory mechanism. We then highlight the significant role of EMP in tumor resistance to chemotherapy and targeted therapy as well as implications of EMP in immune evasion and resistance to immunotherapy. Furthermore, we outline the therapeutic strategies targeting EMP, including small molecule inhibitors, immunotherapeutic approaches, and combination therapies, to support follow‐up clinical research. We also discuss the future perspectives and emerging technologies for deeper study on the heterogeneity characteristics induced by EMP and promising development of novel therapy. This review aims to offer readers a comprehensive and multifaceted elucidation of the complex mechanism and regulation of EMP in cancer for the exploration of potential therapeutic prospects.

## 2 EMT AND MET

### 2.1 EMT and MET processes in cancer biology

**FIGURE 1:**
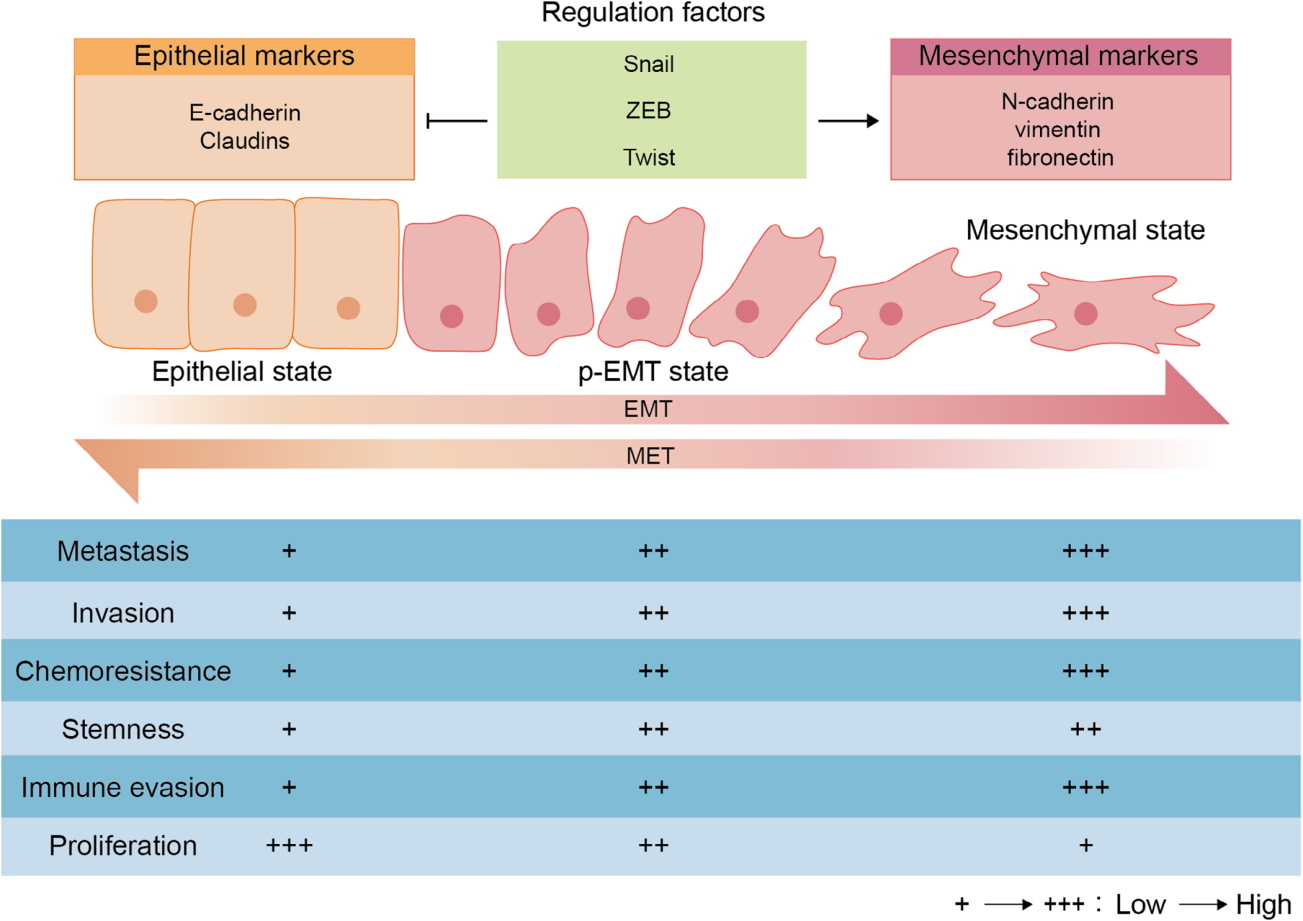
The processes and features of EMT and MET. Tumor cells undergoing EMT exhibit a decrease in epithelial markers and an increase in stromal markers, while cells undergoing MET exhibit the opposite. And these processes are regulated by the key EMT‐related transcription factors such as Snail, ZEB, and Twist. The process of EMP is not a binary process, for tumor cells can move between an epithelial and a mesenchymal state to adapt to various new environments during tumor progression and own different features. EMT, epithelial–mesenchymal transition; MET, mesenchymal–epithelial transition.

One of the vital drivers during tumor deterioration is the ability that malignant tumor cells can migrate from the primary site to potential peripheral environment and distant organs, then forming metastatic lesions. Tumor metastasis is a complex and multistage process, which comprises three stages: (i) invasion in the primary tumor site; (ii) hematogenous spread; and (iii) extravasation and colonization in distant organs.^15^ The dynamic surrounding niche of tumor cells during different metastasis stages impacts the EMP process to better survive and metastasize.^16^, ^17^ The process of EMP is characterized as EMT and MET, which is not a binary process (Figure 1), since tumor cells can transition between an epithelial and a mesenchymal state to adapt to various new environments during tumor progression.^18^

EMT, the main assistor of migration and the process that tumor cells switch from epithelial phenotype to fully or merely partially mesenchymal phenotype with a loss on cell polarity as well as intercellular de‐adhesion, contributes to the tumor cells invasion and migration through the ECM and vascular system.^19^ Epithelial cells with low levels of migration have a compact connection with ECM and high cell–cell adhesion via expression of adherent proteins for example E‐cadherin.^20^ During EMT, more mesenchymal‐like cells with downregulation of E‐cadherin and upregulated mesenchymal markers including N‐cadherin, vimentin and fibronectin exhibit loss of tight connection and become more invasive and motile.^21^ More importantly, p‐EMT state tumor cells that lie in a neutral state between the utterly epithelial and mesenchymal states are observed to be more invasive and higher metastatic with peculiar features such as collective migration, microenvironmental organization, metabolic reprogramming, and immune evasion.^22^, ^23^ The higher p‐EMT levels can drive tumor progression through increasing the stemness and chemoresistance of tumor cells, which are positively associated with lower overall survival and higher mortality.^24^, ^25^

On the contrary, recent research has shown that mesenchymal‐like cells can reacquire epithelial properties through the opposite process, MET, which facilitates the colonization and growth of metastatic colonies via re‐establishing connections to surrounding cells and re‐obtaining cell polarity.^26^ The maintenance of reversible MET process shows that the EMP during tumor deterioration is highly dynamic. The process of MET occurs in metastatic niche, during which tumor cells regain epithelial properties to achieve colonization and growth since distant metastases usually show the similar epithelial structural features pertain to the tissue that they originated from.^27^ MET is often recognized as the re‐expression of E‐cadherin, decrease in mesenchymal properties as well as the formation of metastatic foci. The expression of E‐cadherin is essential for the tumor‐initiating capacity, while mesenchymal features with high ZEB1, ZEB2, and SNAIL can impair this capacity.^28^

### 2.2 Role of EMP in cellular phenotype switching and tumor heterogeneity

As an extremely complex and malignant disease, cancer displays profound heterogeneity that is attributed to the intratumorally phenotypic diversity, which may be explained by the mechanism of cellular plasticity especially EMP.^4^, ^29^ High tumor heterogeneity is considered a significant challenge when treating cancer for it is tightly related to metastasis and therapy resistance.^30^, ^31^ A wide range of stable phenotypic cells are distinct in characteristics of metastasis or escaping therapy.^32^ Moreover, the cellular plasticity can facilitate the dynamic cellular phenotype switching, making tumor cells to better adapt to different environments such as metastatic niche and exposure to distinct therapeutic agents.^33^

EMP plays a vital role in tumor heterogeneity, including both morphological heterogeneity and functional heterogeneity. The morphological heterogeneity is apparent since it can be observed that the morphology of tumor cells that undergo EMT switches from round to fusiform due to the change of their cytoskeleton and generation of vimentin filaments.^34^ With the change of morphology, mesenchymal tumor cells acquire the capacity to invade and migrate. This implies that the switch of cellular morphology can lead to acquisition of various cellular function. More importantly, EMP can bring out more functional heterogeneity of tumor cells that drives deterioration. Tumor cells in different EMT states are distinct in therapy sensitivity. Recent research demonstrates that tumor cells undergoing MET are sensitive to therapy, while cells undergoing EMT are resistant to therapy.^13^ EMP enables tumor cells to switch their phenotypes between drug‐sensitive and ‐resistant phenotypes, resulting in better survival and resistance heterogeneity under treatment.^35^ Besides, EMP is also correlated to the metabolic heterogeneity of tumor, which participates in the process of tumor cells proliferation and migration. Oxidative phosphorylation (OXPHOS) and glycolysis are two main sources that tumor cells gain energy. Recent study shows that OXPHOS is negatively correlated with epithelial phenotypes, while glycolysis is positively correlated with mesenchymal phenotypes.^36^, ^37^ Moreover, EMP also plays a potential role in acquiring and inducing the dormant phenotype of tumor.^38^

In conclusion, EMT and MET are the reversible process in tumor cells and are crucial for the acquisition of various malignant features of tumor cells that drive tumor deterioration. Therefore, it is necessary to understand the mechanisms that regulate EMP, which can provide valuable insights into the regulation of EMP in future.

## 3 TRANSCRIPTIONAL REGULATION OF EMP

Transcriptional programs predominantly determine the cell identity and are driven by TFs that can participate in the expression and silence of various specific genes for the maintenance of cellular phenotypes.^39^ EMT/MET process is initiated as response to multiple specific stimuli in surrounding environments such as hypoxia, inflammation and oxidative stress.^38^, ^40^ When faced with stress conditions, EMT signaling is activated and mediated via EMT‐TFs to modulate the EMT‐related genes and subsequently impact on the cellular phenotype.^41^ Significantly, EMT‐TFs coordinate with several epigenetic mechanisms including deoxyribonucleic acid (DNA) methylation and histone modification to involve in phenotypic transformation of EMP.^40^ Here, we summarize the role of key EMT‐TFs and the main epigenetic modifications regulating EMP.

### 3.1 Role of EMT‐TFs in EMP regulation

TFs are one of the vital actors with DNA binding domains in regulating the gene expression through directly binding to specific regulatory DNA sequences (cis‐regulatory genomic elements), regulating ribonucleic acid (RNA) polymerase II activity and subsequently impacting on important biological processes such as cellular fate and plasticity.^42^, ^43^ It has been reported that deregulation of TFs could contribute to tumorigenesis and appearance of other diseases.^44^ TFs have been found to be responsible for phenotypic transformation, including mutual transformation between various malignant phenotypes.^45^ The initiation of EMT‐related gene expression program involves a broad range of TFs, in which Snail, ZEB, and Twist are thought to be the core EMT‐TFs that orchestrate inclusive gene expression responses.

#### 3.1.1 Snail

Snail family members include Snai1 (Snail), Snai2 (Slug), and Snai3 (Smuc). The expression of Snail and Slug has been demonstrated to be the biomarkers of tumor metastasis, and the overexpression of these two EMT‐TFs is associated with poorer prognosis of tumor.^46^, ^47^ Snai1 is found to be encoded by the SNAI1 gene, the expression of which can lead to direct suppression of the Cadherin1 (CDH1) gene encoding E‐cadherin. Snail is recognized as a zinc‐finger transcriptional repressor for its inhibitory functions. And the structure of Snail consists of several domains, including an N‐terminal SNAI1/GFI (SNAG) domain, four C‐terminal C2H2 zinc‐finger domains, a nuclear export signal domain, and a protein destruction box domain. The SNAG domain of Snail is thought to be required for the inhibition of E‐cadherin.^48^ Slug comprises the N‐terminal and C‐terminal domains similar to Snail as well as an exclusive SLUG domain. The SNAG and SLUG domains within Slug is considered to be essential for the suppression on the CDH1 gene promoter.^49^ Apart from E‐cadherin, the gene of other epithelial markers can also be suppressed by Snail and Slug, including claudin, occludin, and desmoplakin, while expression of mesenchymal markers such as N‐cadherin, vimentin, and metalloproteinases (MMPs) can be increased by Snail and Slug.^50^, ^51^

The level of Snail can be increased by diverse external signals and mediate the signaling pathways inducing EMT. Epidermal growth factor receptor (EGFR), a transmembrane receptor tyrosine kinase, plays a significant role in the progression of various tumors. EGFR can be activated by epidermal growth factor (EGF) and then lead to the promotion on tumor cells proliferation and migration via inducing EMT. Recent study shows that EGF‐activated EGFR can enhance the phosphorylation of Akt and glycogen synthase kinase‐3β (GSK‐3β) and subsequently the upregulation of Snail to induce EMT.^52^ Besides, the abnormal environmental molecule can also upregulate Snail to promote EMT process. For instance, the accumulated lactate generated from metabolic reprogramming can increase the expression of the cell surface receptor CD38 via the Hippo–TAZ pathway. And then the increased CD38 activate adenosine–A2AR signaling to enhance the Snail expression through the Akt/GSK‐3β pathway, finally resulting in the progression of EMT.^53^

#### 3.1.2 ZEB

The ZEB family members including ZEB1 and ZEB2 are the core EMT‐TFs that mediate EMT process and are associated with the progression of various malignant cancers such as invasion, resistance, and metastasis. The ZEBs structure contains a homeodomain in its midst and other protein binding domains are contained in the ZEBs structure, including the SMAD interaction domain that mediates the regulation of transcription induced by TGF‐β and bone morphogenetic proteins signaling, a zinc finger domain, a coactivator binding domain, a CtBP interaction domain as well as a p300‐CREB‐binding protein (CBP)‐associated factor binding domain. The aforementioned domains are thought to induce EMT as well as contribute to the metastasis and other malignant events in tumor.^54^

The high expression of ZEB1 is involved in the upregulation of cytoplasmic and nuclear N‐cadherin, while the high level of ZEB2 is correlated with the upregulation of cell membrane N‐cadherin.^55^, ^56^ There exists a negative feedback circuit between miR‐200 and ZEB1/2 and this feedback circuit is vital for the phenotypic switch in different states of EMP. MiR‐200 can target the genes dynamically regulating the cytoskeleton and downstream of TGF‐β and EGFR signaling to impact on EMT.^57^ During EMT process, the upregulated ZEB1/2 can directly bind the miR‐200 promoter to suppress its transcription. On the contrary, ectopic expression of miR‐200 such as miR‐200a and miR‐200c can repress ZEB1/2 directly to induce MET in mesenchymal cancer cells.^41^ Recent research using molecular binding data as well as mathematical modeling ulteriorly demonstrates that the miR200/ZEB circuit is vital for the retention of p‐EMT states and regulation of EMP.^58^

#### 3.1.3 Twist

Twist family members comprise Twist‐1 and Twist‐2, both of which are found to be upregulated and induce EMT process in tumor tissues.^59^, ^60^ The mechanisms that Twist regulates EMT involve a variety of avenues, including regulation of EMT‐associated genes and downstream effectors.^61^ Synaptotagmin7 (SYT)7, one of the synaptic binding protein gene family members associated with migration of tumor cells, is upregulated in tumor tissues and promote the tumorigenesis and metastasis via inducing EMT. Recent research demonstrates Twist‐1 significantly alters the expression of SYT7, which is recognized as the downstream gene of Twist‐1 inducing EMT.^62^ Besides, Twist can transcriptionally induce the expression of Protease‐activated receptor‐1 (PAR1) directly, which is the upstream signal in Hippo pathway. Subsequently, the Twist‐mediated PAR1 activation contributes to the induction of Yes‐associated protein (YAP)/transcriptional coactivator with PDZ‐binding motif (TAZ) and finally trigger of EMT.^63^

### 3.2 Epigenetic modifications influencing EMP

In addition to TFs, epigenetic modifications also control gene expression, which are thought to be a bridge connecting the alteration in the outer surroundings and gene expression regulation.^64^ Epigenetic factors, including DNA methylation and histone modifications, are thought as a main driver of phenotypic diversification in cancer cells, revealed by recent studies using sequencing technologies.^65^ Many studies suggest that epigenetic changes are able to induce EMT of cancer cells to enhance the invasion, metastasis, and resistance in cancer.^66^, ^67^, ^68^ Due to the reversibility of epigenetic changes, here we summarize the role of stereotypical epigenetic factors, DNA methylation, and histone modifications, in regulating EMP to better understand the dynamic phenotypic switch of EMP.

#### 3.2.1 DNA methylation

DNA methylation denotes the transfer of a methyl group to 5th carbon atom position on cytosine in CpG dinucleotides, which leads to 5‐methylcytosine formation. The DNA methylation process is catalyzed by a series of enzymes, including several members of DNA methyltransferase (DNMT) family: DNMT1, DNMT3a, and DNMT3b.^69^ Among these DNA methyltransferase enzymes, DNMT3a and DNMT3b can generate the de novo DNA methylation, while DNMT1 is responsible for the maintenance of DNA methylation.^70^ DNMT1 is found to be induced by reactive oxygen species and can contribute to the promoter‐CpG‐island methylation of CDH1. The increased DNMT1 work with EMT‐TFs Snail and Slug to synergistically inactivate the CDH1 gene, resulting in the reduction of E‐cadherin as well as promotion on EMT.^71^ Besides, DNMT3b is upregulated in cancer cells with invasive phenotype and also inhibit the expression of E‐cadherin.^72^ Moreover, recent research suggests that the upregulation of DNMTs leads to the downregulation of miR‐29c‐3p via dictating promoter CpG sites’ hypermethylation to mediate the EGFR/Akt as well as Wnt/β‐catenin signalings and finally induce the EMT process.^73^

#### 3.2.2 Histone modifications

Histone modifications are another key factor that modulates the expression of gene and play a significant role in carcinogenesis.^74^ Histone modifications include methylation, acetylation, and other modifications on the histones that wrap DNA to maintain DNA structure. Irregular histone modifications have been observed in tumor progression and metastasis. Wolf‐Hirschhorn syndrome candidate 1 (WHSC1), a histone methyltransferase that can induce the histone H3 lysine 36 methylation to regulate transcription of gene, is increased in malignant tumor and correlated with tumor growth and metastasis. WHSC1 is found to promote the activation of transforming acidic coiled‐coil containing protein 3, the expression of which can upregulate phosphatidylinositol‐3‐kinase (PI3K)/Akt signaling and induce EMT process.^75^ In addition, histone methyltransferase SET and MYND domain containing 2 (SMYD2) epigenetically modulates SMAD3 expression and the knockdown of SMYD2 attenuates the process of EMT.^76^ Histone deacetylase (HDAC) is one of the main players of histone acetylation. Osteopontin (OPN), a canonical cancer marker protein and also the downstream of TGF‐β, has several splicing isoforms including OPNa, OPNb, and OPNc, among which the OPNc is recognized as functional splicing isoform that activate EMT signaling. HDAC is reported to mediate the splicing of OPNc from the full‐length isoform OPNa and the increase of OPNc expression to induce EMT.^77^

Taken together, transcriptional regulation is essential for the maintenance of cellular phenotypes. EMT‐TFs coordinate with epigenetic mechanisms to modulate the expression of EMP‐related genes, thereby influencing the phenotypes of tumor cells. The changing transcriptional regulation in tumor cells explains the dynamic phenotypic switching during tumor development.

## 4 SIGNALING PATHWAYS REGULATING EMP

Signaling pathways involving regulation of EMP include TGF‐β, Wnt/β‐catenin, Notch, and other key signaling pathways. These signaling pathways are considered as messengers between molecular mechanisms related to EMP, such as hypoxia and inflammation, and induction of EMT‐TFs to impact on the expression of EMP‐related genes, thereby impacting on the phenotypic variation of tumor cells.^78^ Here, we focus on the research progress on the main signaling pathways regulating EMP.

### 4.1 TGF‐β signaling pathway and its role in inducing EMT

**FIGURE 2:**
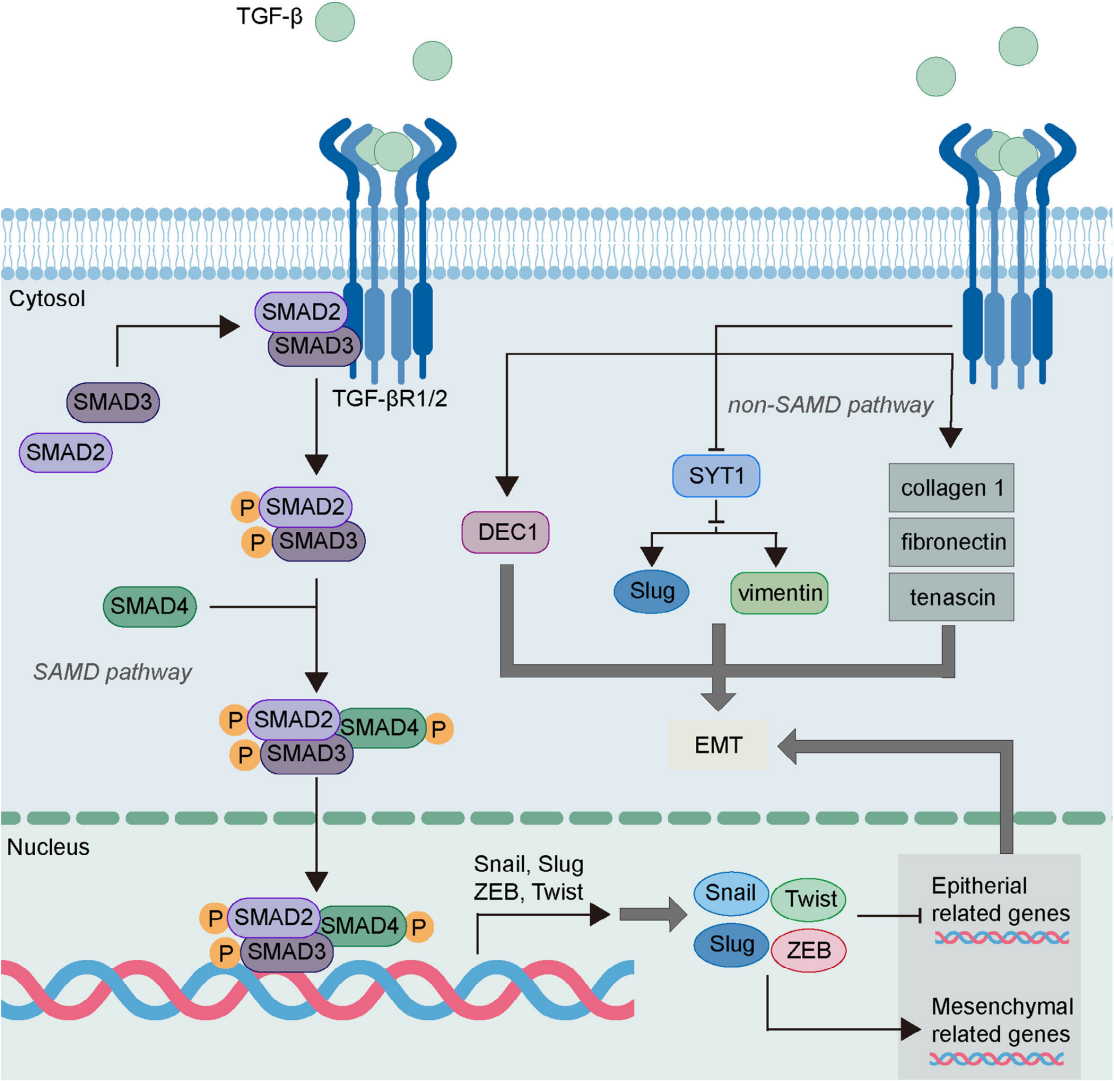
Regulation of EMT via TGF‐β‐induced SMAD and non‐SMAD pathways. The binding of TGF‐β and its receptor TGF‐βR1/2 leads to the formation and nuclear translocation of Smad2/3/4 complex, which promote the expression of EMT‐TFs. Besides, TGF‐β also regulates EMT via non‐SMAD pathways. DEC1, differentiated embryo‐chondrocyte expressed gene 1; EMT, epithelial–mesenchymal transition; SYT1, synaptotagmin; TGF‐β, transforming growth factor‐β; TGF‐βR1/2, TGF‐β cell surface receptors type I/II.

TGF‐β is the critical player and one of the first EMT inducers that regulate the EMP of tumor cells. The highly expressed TGF‐β is correlated with the promoted EMT process and poor prognosis of tumor.^79^, ^80^ Intriguingly, the different TGF‐β concentrations determine the diverse transition of epithelial, p‐EMT, and mesenchymal states in tumor cells via influencing the expression of EMT‐TFs, E‐cadherin, and vimentin.^12^ TGF‐β can activate SMAD pathways and non‐SMAD pathways, both of which are significant for the regulation of EMP (Figure 2).

The signal transduction of TGF‐β depends on the SMAD family including SMAD2, SMAD3 and SMAD4. The binding of TGF‐β and the corresponding TGF‐β cell surface receptors, type I (TGF‐βR1) and type II (TGF‐βR2) receptors, contributes to the formation of tetrameric complexes that activate downstream SMAD2 and SMAD3 to form heteromeric complexes with SMAD4.^81^ Subsequently, the activated SMAD complexes translocate into the nucleus and upregulate the expression of EMT‐TFs.^38^ More mechanisms of TGF‐β/SMAD pathways have been studied in recent research. Olfactomedin 2 (OLFM2), a glycoprotein that is associated with cell adhesion and differentiation, is reported to be elevated in colorectal cancer and can promote the EMT and invasion of cancer cells.^82^, ^83^ The highly expressed OLFM2 can lead to reduced E‐cadherin expression and enhanced N‐cadherin and vimentin expression, and this can be explained by its role in upregulating TGF‐βR1 expression and enhancing the phosphorylation of SMAD2 and SMAD3.^82^ Besides, the TGF‐β/SMAD2/3 pathway induced by the deficiency of Parkin, an E3 ubiquitin ligase found to be decreased in cancer, can induce migration and EMT in cancer cells via activating the transmembrane protein with EGF‐like and two follistatin‐like domains 1 gene that can act as a tumor‐promoting gene.^84^ Moreover, TGF‐β/SMAD3 is found to upregulate Snail expression, contributing to the activation of RHOA, which is critical for cell motility and subsequent induction of stress fiber formation as well as cell morphological changes.^85^

Besides SMAD pathways, non‐SMAD pathways also mediate the EMP regulation of TGF‐β. In prostate cancer, TGF‐β can trigger the tumorigenic alterations of ECM, leading to acquisition of a migratory phenotype via initiating EMT.^86^ In colorectal cancer, TGF‐β could reduce the expression of SYT1, a family of structurally related proteins that can inhibit EMT via downregulating the Slug and vimentin expression.^87^ In addition, TGF‐β promotes the expression of differentiated embryo‐chondrocyte expressed gene 1 (DEC1), a helix‐loop‐helix (bHLH) TF, and the dysregulated DEC1 is found to participate in the TGF‐β‐mediated EMT, resulting in tumor metastasis.^88^, ^89^

### 4.2 Wnt/β‐catenin pathway in EMP regulation

**FIGURE 3:**
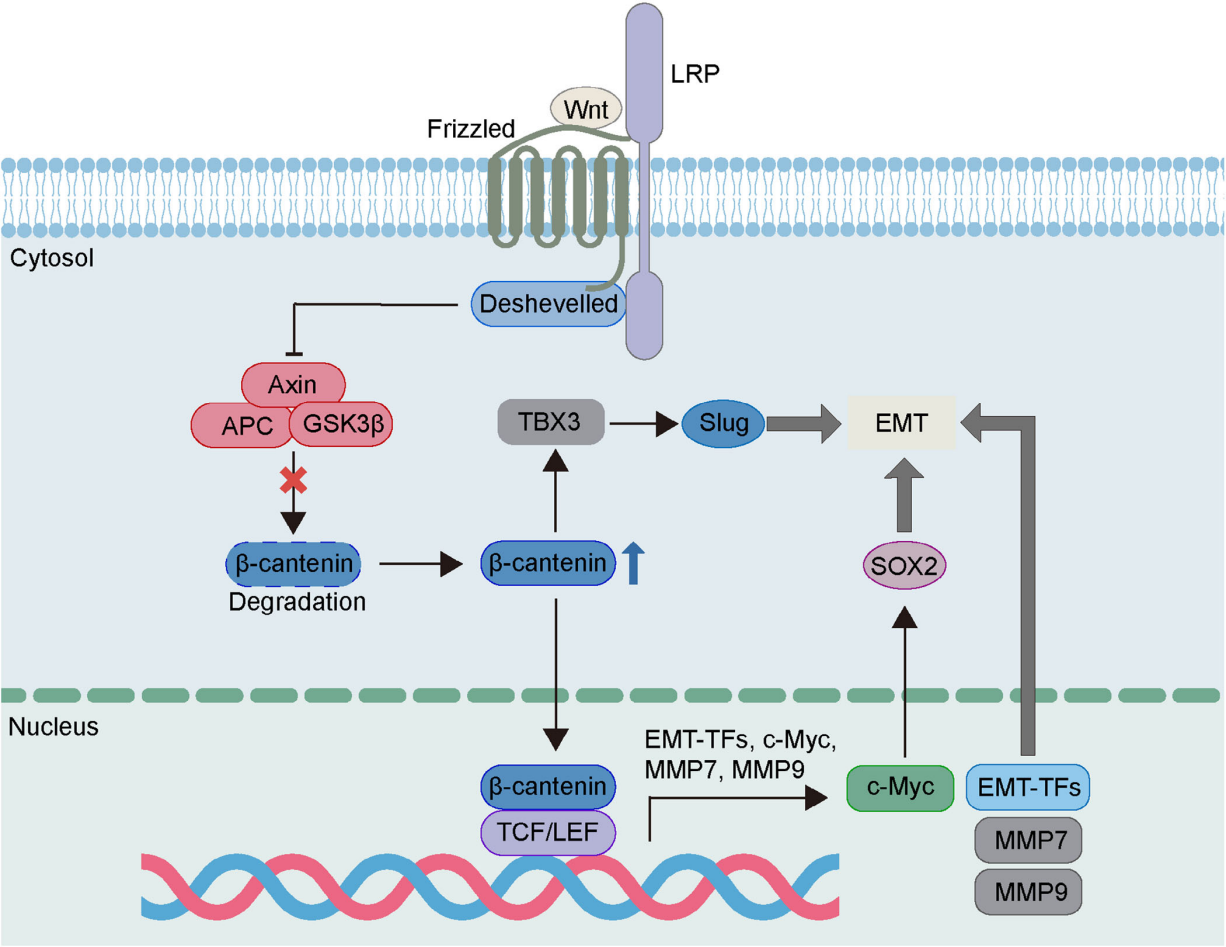
Regulation of EMT via Wnt//β‐catenin signaling. Binding of Wnt and Frizzled leads to the recruitment of coreceptor LRP5/6, which activates Deshevelled. The activated Deshevelled inhibit the β‐catenin‐destruction complex comprised of GSK3β, Axin (scaffolding protein) and APC, which enhances the degradation of β‐catenin, to stabilize the β‐catenin in cytoplasm. Then, the elevated β‐catenin is transferred into nucleus and binds to transcription factor TCF/LEF, eventually leading to activation of target genes that regulate EMT. EMT, epithelial–mesenchymal transition; EMT‐TFs, EMT‐transcription factors; GSK3β, glycogen synthase kinase 3‐β; MMP7, metalloproteinase7; MMP9, metalloproteinase9; SOX2, sex determining region Y‐box 2; TBX3, T‐box transcription factor 3; TCF/LEF, T cell factor/lymphoid enhancer factor.

Wnt/β‐catenin is an evolutionary conserved signaling critical for embryonic development, adult tissue homeostasis and other diverse biological processes.^90^, ^91^, ^92^ The dysregulated Wnt/β‐catenin is found to participate in the progression of various cancers such as initiation, metastasis and recurrence.^93^ Wnt signaling is a crucial regulator of EMP, which can promote the mesenchymal phenotype transformation of cancer cells. β‐Catenin is a significant component of cytoskeleton and the key player of Wnt signaling. When Wnt ligands bind to Frizzled protein receptors, the degradation of β‐catenin will be repressed, and this contributes to the increase of β‐catenin at cytoplasm.^92^, ^93^ Subsequently, the elevated β‐catenin is transferred into nucleus and then binds to TFs such as T cell factor/lymphoid enhancer factor (TCF/LEF), eventually leading to activation of target genes including Snail, ZEB, Twist, MMPs, and c‐Myc, a main carcinogenic driver of tumor progression (Figure 3).^93^, ^94^, ^95^, ^96^ The c‐Myc induced by Wnt/β‐catenin is reported to regulate the expression pf sex determining region Y‐box 2 (SOX2), a TF crucial for the cellular stemness, resulting in the promotion of EMT and metastasis.^97^ Besides, Wnt/β‐catenin can upregulate the T‐box TF 3 (TBX3) expression as well as enhance EMT simultaneously, and this may be associated with the function of TBX3 in upregulating Slug expression.^98^ Interferon‐induced protein with tetratricopeptide repeats 1 (IFIT1), an inflammation‐related protein dysregulated in cancer, is associated with the migration and invasion of pancreatic cancer cells. And it has been reported that Wnt/β‐catenin signaling can mediate the tumor‐promoting function of IFIT1 via activating EMT.^99^ Epigenetic factor lysine demethylase 5B (KDM5B) is an oncogene aberrantly expressed in various cancers and plays a crucial role in lymph node metastasis and tumor recurrence. Recent study suggests that KDM5B can activate Wnt/β‐catenin signaling to promote EMT and metastasis in squamous cell carcinoma of the head and neck.^100^

### 4.3 Notch signaling and its role in EMP

**FIGURE 4:**
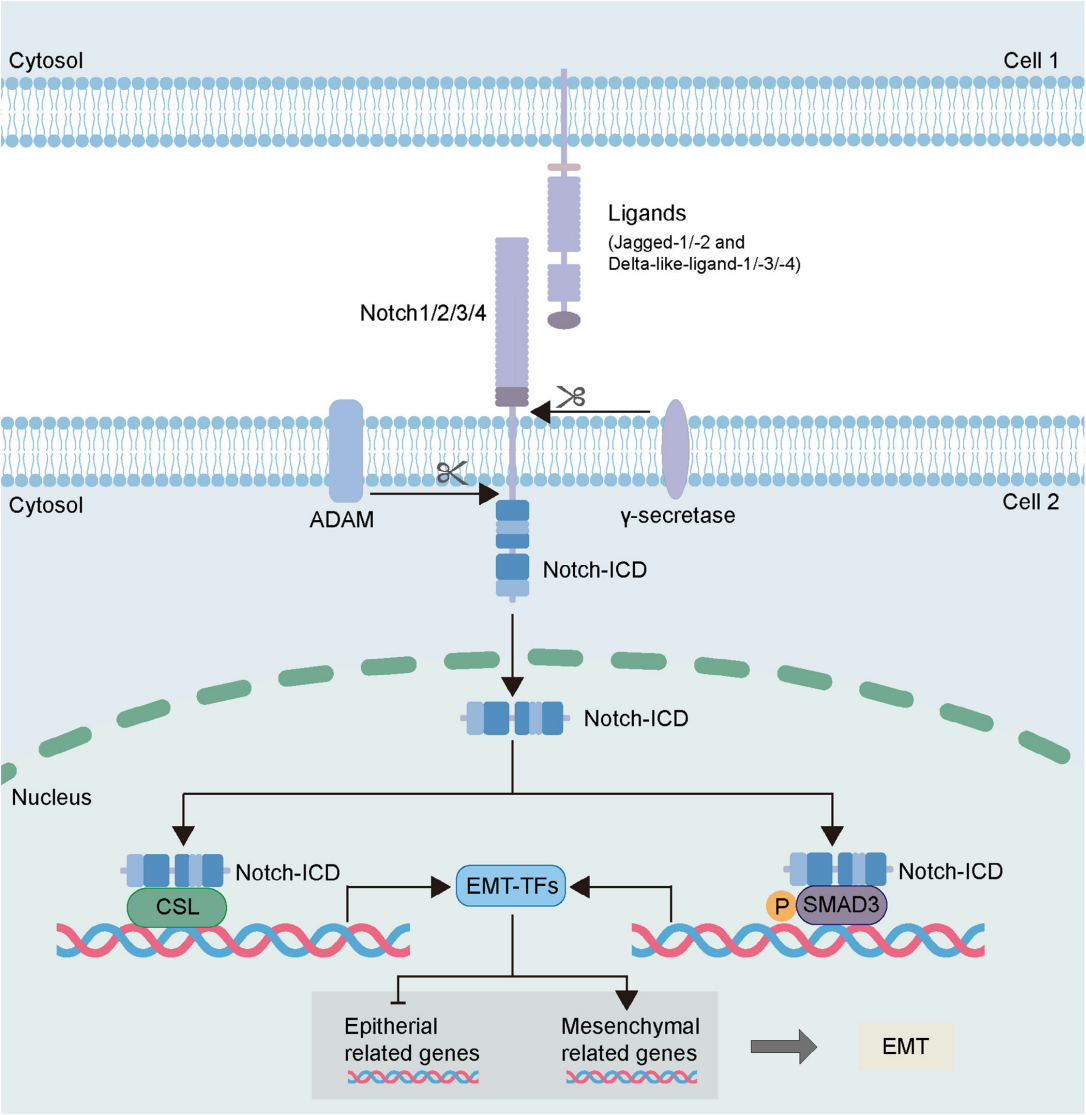
Regulation of EMT via Notch signaling. The Notch receptor is activated upon binding to the Notch ligand, leading to the Notch receptor cleavage by ADAM and γ‐secretase complex. This cleavage contributes to the release of Notch‐ICD and its translocation into the nucleus. Then, the Notch‐ICD interact with CSL or phosphorylated SMAD to activate target EMT‐TFs genes. ADAM, a disintegrin and metalloproteinase; CSL, CBF‐1/suppressor of hairless/Lag1; EMT, epithelial–mesenchymal transition; EMT‐TFs, EMT‐transcription factors; Notch‐ICD, Notch‐intracellular domain.

Notch is one of the oldest identified signaling involved in various critical biological processes such as the EMT process in embryonic development and tumor progression.^101^ Notch signaling will be activated upon the binding of Notch receptors (Notch1‐4) with Notch ligands (Jagged‐1, ‐2, and Delta‐like‐ligand‐1, ‐3, ‐4), leading to the cleavage of the receptors as well as the release and translocation of the Notch‐intracellular domain (ICD) into the nucleus, which acts to activation of Notch downstream genes (Figure 4).^102^, ^103^ The components of Notch signaling are reported to be commonly overexpressed in cancers and play a significant role in regulating tumor progression. The increased Notch1 and Jagged‐1 is associated with tumor metastasis and has the ability to enhance the migration and EMT of tumor cells.^104^, ^105^, ^106^ Besides, the tumor with high Notch3 expression is found to develop rapidly with poor prognosis.^107^ The mechanisms of Notch signaling in promoting tumor progression via impacting on EMT have also been studied in recent research. The Jagged‐1‐activated Notch signaling can repress the E‐cadherin expression via activating Slug to induce EMT.^108^, ^109^ The Slug promoter can be activated by the Notch‐ICD translocated into nucleus, and the knockdown of Slug reverses the EMT process induced by Jagged1/Notch1.^110^ The upregulated cysteine‐rich 61 (CYR61), a secreted protein involved in tumor metastasis, is reported to induce expression of EMT‐TF Twist, vimentin, and N‐cadherin. Recent research suggests the CYR61 in breast cancer can mediate the EMT induced by Notch1, indicating CYR61 acts as downstream of Notch in regulating EMT.^111^ Additionally, Hes1 is also the downstream of Notch. The expression of Notch1, Notch2, and Notch3 inhibited by ginsenoside Rg3, a bioactive ginseng compound that exhibits antitumor effects, can lead to the repression of EMT via reducing Hes1 expression.^112^ Interestingly, recent research indicates that Notch signaling participates in the EMT induction mediated by TGF‐β, and this process is mediated via the TGF‐β‐triggered phosphorylated SMAD3.^113^, ^114^

### 4.4 Other key signaling pathways involved in EMP

Apart from aforementioned signaling pathways, there are other developmental signaling pathways orchestrate EMP such as PI3K/Akt/mammalian target of rapamycin (mTOR), Hedgehog, and Hippo. PI3K/Akt/mTOR signaling is one of the most critical regulators of various cellular process, particularly in controlling cell cycle and tumor progression. Aberrantly activated PI3K/Akt/mTOR signaling is involved in the metastasis of cancers, while inhibition of PI3K/Akt/mTOR can lead to EMT reverse and reduced metastasis.^115^, ^116^ Recent research suggests that PI3K/Akt/mTOR induced by glutathione peroxidase 2, which is an antioxidant enzyme associated with tumor metastasis, can upregulate Snail to promote EMT process and metastasis in non‐small cell lung cancer.^117^ Phosphoglycerate mutase 1 (PGAM1) is a glycolytic protein abnormally expressed in cancers that can enhance EMT in pancreatic cancer cells via regulating Wnt/β‐catenin pathway. PI3K/Akt/mTOR are found to upregulate the PGAM1 expression in pancreatic cancer cells to induce EMT, contributing to high metastatic potential of pancreatic ductal adenocarcinoma.^118^ Hedgehog signaling is another significant pathway involved in EMT, which can activate TFs of glioma (GLI) family. The activated Hedgehog/GLI1 signaling has been shown to enhance EMT process in various cancers.^119^, ^120^ Forkhead box S1 (FOXS1) is a member of FOX TFs that can modulate EMT and is correlated with poor prognosis of tumor.^121^, ^122^ FOXS1 can inhibit the ubiquitination of GLI1 to upregulate Hedgehog/GLI1 signaling, which leads to tumor metastasis via inducing EMT.^122^ Besides, Hedgehog/GLI is reported to act with Notch signaling in colorectal cancer cells to drive EMT to promote tumor invasiveness.^123^ Moreover, Hippo signaling pathway also participates in the modulation of EMT, the main effector downstream of which is the YAP. Recently, glycine decarboxylase (GLDC) has been recognized as a tumor‐promoting gene upregulated in various cancers. The Hippo/YAP signaling pathway is reported to mediate the metastasis induced by GLDC‐activated EMT in colorectal cancer.^124^ Additionally, Hippo/YAP signaling is involved in the ovarian cancer metastasis enhanced by Piezo‐type mechanosensitive ion channel component 1, a gene responsible for EMT process.^125^ In summary, the signaling pathways regulating EMP are diverse and may participate in the signaling transduction of each other, offering potential intervention targets for the treatment of EMP.

## 5 DYNAMIC MICROENVIRONMENTAL INFLUENCES ON EMP

**FIGURE 5:**
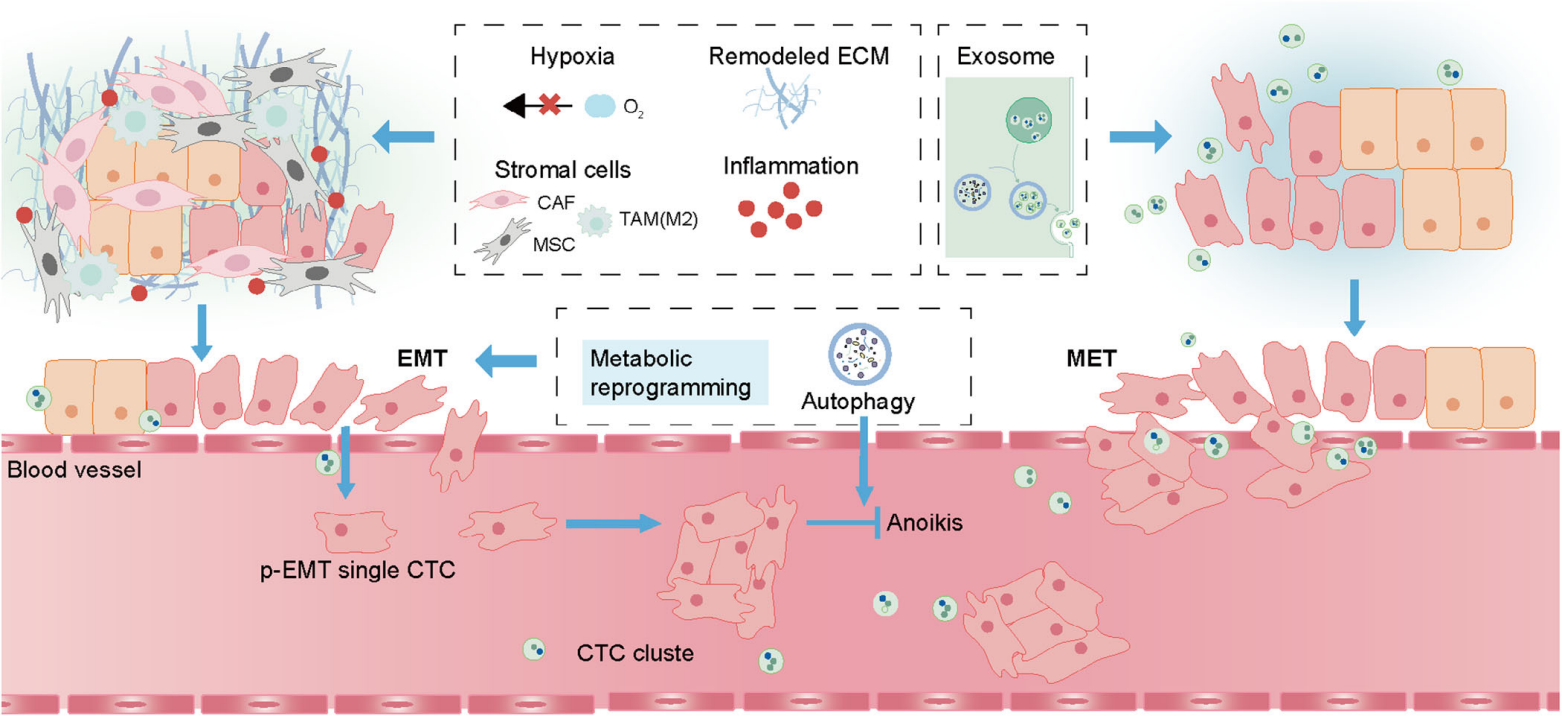
The dynamic microenvironmental influences on EMP during tumor progression. The extracellular EMP‐driven factors including hypoxia, inflammation, remodeled ECM, and stromal cells act with the stress conditions‐induced metabolic reprogramming and autophagy to impact on the EMP states of tumor cells via inducing EMT in the primary niche. In the vascular niche, autophagy can support the p‐EMT CTCs to resist anoikis induced by ECM detachment. Besides, exosomes in the premetastatic niche can promote the MET of tumor cells to form colonization. CAF, cancer‐associated fibroblast; CTCs, circulating tumor cells; ECM, extracellular matrix; EMP, epithelial–mesenchymal plasticity; EMT, epithelial–mesenchymal transition; MET, mesenchymal–epithelial transition; MSC, mesenchymal stem cell; TAM, tumor‐associated macrophage.

Besides the classical signaling pathways regulating EMP, EMP is also regulated by various extracellular molecular mechanisms in the interacting dynamic microenvironments and the microenvironment‐induced intracellular molecular mechanisms synergistically. The highly complex and versatile character of EMP process is tightly related to the dynamic surrounding niche including primary niche, vascular niche, and premetastatic niche, which collectively afford the deterioration of tumors. With changes in the environment, the extracellular EMP‐driven factors such as hypoxia, inflammation, remodeled ECM, stromal cells, and EVs will impact on the EMP states of tumor cells via regulating EMP‐related signaling pathways. Meanwhile, the stress conditions in environment will also lead to the activation of intracellular mechanisms that regulate EMP, including metabolic reprogramming, domesticated autophagy, and anoikis (Figure 5).

### 5.1 Extracellular molecular mechanisms influencing EMP

#### 5.1.1 Hypoxia

Hypoxia, which is related to the increased risk of metastasis, is recognized to be an essential feature of the tumor microenvironment due to the imbalance between the oxygen supply and demand and insufficient vascularization.^126^ The tumor cellular response to hypoxia including induction of EMT is generally mediated by hypoxia‐inducible factors (HIFs).^127^, ^128^ The mechanisms of hypoxia‐induced HIFs in regulating EMP have been studied in recent research. In esophageal squamous cell carcinoma, HIF‐1α is found to activate DNA Polymerase iota expression via binding to its gene promoter, which in turn regulates EMT‐related genes and initiates EMT via stabilizing HIF‐1α.^129^ Besides, increased HIF‐1α can act as a TF to enhance adenosine 5'‐triphosphate (ATP)‐citrate lyase activation in hypoxia conditions, which can promote EMT in gastric cancer and is tightly associated with tumor metastasis.^130^ Nidogen‐1 (NID1), a basement membrane component reported to be upregulated in tumor occurrence, can enhance the invasion and metastasis of tumor. In salivary gland adenoid cystic carcinoma, HIF‐1α induces the overexpression of NID1, which contributes to EMT and metastasis via activating the PI3K/Akt pathway.^131^ Significantly, HIF‐1α can promote the Hedgehog TF GLI1 expression, and subsequently the overexpressed GLI1 mediates the invasion, migration, and EMT induced by HIF‐1α.^132^ Additionally, hypoxia‐induced overexpression of HIF‐3α upregulates miR‐630 expression, which promotes the EMT‐mediated metastasis in cervical cancer cells.^133^

#### 5.1.2 Inflammation

Inflammation is a well‐known driver of cancer progression in the primary niche since inflammatory responses mediated by inflammatory cytokines such as interleukins (ILs), tumor necrosis factor‐α (TNF‐α), and nuclear factor‐kappa B (NF‐κΒ), are involved in almost all stages of cancer as well as EMT.^134^, ^135^ IL‐1β and IL‐1 receptor antagonist (IL‐1RA) are found to be upregulated in colorectal cancer. The upregulation of IL‐1β/IL‐1RA axis activates autophagy, which mediates the downregulation of E‐cadherin and upregulation of N‐cadherin, vimentin, and Snail to regulate EMT in human colon cancer cells.^136^ RHOJ, a member of RHO guanosine triphosphatases family, is observed to be overexpressed in EMT‐subtype gastric cancer with decreased CDH1 expression and high mesenchymal genes expression. Recent research suggests that RHOJ can activate the IL‐6/ signal transducer and activator of transcription 3 signaling, resulting in EMT‐induced invasion and metastasis of gastric cancer.^137^ NF‐κΒ is recognized as the downstream of IL‐17A and can lead to activation of NLRP3, one of the most representative inflammasomes. Recent research reports that IL‐17A activates the NLRP3 inflammasome, which mediates the EMT process in lung cancer.^138^ Besides, the repression of NF‐κΒ is found to repress TNF‐α and subsequently EMT in colorectal cancer cells.^139^ C–X–C motif chemokine 10 (CXCL10) is a proinflammatory cytokine that can interact with CXCR3 receptor to stimulate tumor growth and metastasis. It is demonstrated that TNF‐α can activate CXCL10/CXCR3 axis to upregulate PI3K/Akt pathway, contributing to increased Snail expression and eventually EMT in colon cancer cells.^140^

#### 5.1.3 Remodeled ECM

ECM is a dynamic network scaffold in tumor microenvironment, which comprises structural proteins (collagens), proteoglycan and glycoproteins (hyaluronic acid), and cell‐adhesion proteins (fibronectin). The signaling driven by ECM depends on the interactions of ECM components with receptors in the cell surface as well as on mechanosensing.^141^ The abnormal changes in composition of ECM will lead to activate specific receptors such as CD44 on tumor cells and high matrix stiffness, both of which can initiate EMT process via upregulating EMT‐related signaling pathways.^142^, ^143^ ECM is constantly remodeled for the adaptation as well as maintenance of tissue homeostasis, while this process is dysregulated during the development of cancer.^144^ The components of ECM can be remodeled by several mechanisms, including MMPs‐mediated cleavage, lysosomal degradation and the synthesis of ECM molecules.^145^, ^146^, ^147^ MMPs are the main matrix degrading proteolytic enzymes that can remodel ECM and promote EMT to facilitate tumor progression and metastasis.^148^ Overexpression of MMP2 and 9, which can proteolytically cleave the epithelial markers E‐cadherin, has been correlated to the induction of EMT as well as increased invasion of tumor cells.^149^, ^150^ Other than the degradation of ECM, the abnormal ECM components also participate in the increasing stiffness of ECM. For instance, abnormal collagen cross‐linking, the most significant protein in the ECM produced by cancer‐associated fibroblasts (CAFs), increases the stiffness of the tumor matrix, and the increasing matrix stiffness enhances the nuclear translocation of EMT TF Twist1.^143^, ^151^

#### 5.1.4 Stromal cells

Stromal cells embedded in the ECM consist of various cell types, such as CAFs, mesenchymal stem cells (MSCs) and inflammatory cells.^152^ Stromal cells in the primary surrounding niche can impact on the ECM components (as mentioned above) and interact with tumor cells to drive EMT process.^153^ CAFs, as a major stromal component thought to be the primary source of EMT signaling, play a pivotal role in tumor progression by remodeling ECM, secreting cytokines and growth factors.^154^, ^155^, ^156^ Besides, high levels of autophagy in CAFs are found to mediate the secretion of high mobility group box 1, which subsequently upregulates the expression of MMP2, MMP9 as well as Twist to enhance EMT process. MSCs are another type of stromal cells that can induce EMT in tumor cells via paracrine signals, including secretion of cytokines and exosomes.^157^, ^158^ For instance, the paracrine stimulation from platelet‐derived growth factor secreted by MSCs promotes the EMT process of ovarian cancer stem cells.^159^ Moreover, MSC‐derived exosomes enriched with miR‐100 have been observed to significantly inhibit EMT process in colorectal cancer cells.^160^ Besides noninflammatory cells, inflammatory cells also participate in the process of inducing EMT, specifically the tumor‐associated macrophages (TAMs), which are enriched in the peritumoral stroma. Being stimulated by the different surrounding microenvironment, two types of macrophages can be polarized to with different states and functions of macrophage activation, including classically activated (M1) and alternatively activated (M2) phenotypes.^161^ Notably, TAMs in most solid tumors harbor the M2‐like phenotype, which exhibit the ability to promote EMT of tumor cells via multiple signaling pathways including C‐C motif chemokine ligand 22 (CCL22)/C‐C motif chemokine receptor 4 (CCR4) and CCL2/Akt/β‐catenin.^162^, ^163^ In addition, the suppression of M2‐like TAMs is found to impair the migration and EMT in cervical cancer via regulating the TGF‐β/SMAD2/3 signaling pathway.^164^

#### 5.1.5 EVs

After initiating EMT, tumor cells intravasate into the vascular system and become circulating tumor cells (CTCs) that exhibit p‐EMT state.^165^, ^166^ According to the “Seed and Soil” hypothesis, the p‐EMT state CTCs in the vascular niche are the seeds seeking the fertile soil (premetastatic niche) for the colonization and growth to form metastatic colonies in distant organ.^167^, ^168^ EVs that travel to distant organs may be considered as the “fertilizer” of metastatic soil, since it can establish the premetastatic niche to induce MET.^169^, ^170^ EVs are the cell‐derived membranous vesicles that contain the miRNA and protein with ability to promote tumor metastasis, which can be commonly divided into several main types: exosomes, microvesicles, and apoptotic bodies.^170^, ^171^ EVs originate from being secreted by plasma membrane (PM) budding or endo‐lysosomal pathways: inward budding of the PM (early endosomes), maturation of early endosomes (late endosomes) forming intraluminal vesicles, and subsequently multivesicular bodies, which fuse with the PM to release exosomes.^172^ The exosomes derived from tumor cells exhibit significantly upregulated specific microRNAs expression such as miR‐200 family, which is responsible for the poor prognosis of tumor.^173^, ^174^ The miR‐200 family can inhibit the expression of ZEB1 and ZEB2 to maintain E‐cadherin expression in EMT cells, representing the process of MET.^175^, ^176^ In addition, upregulated miR‐200 family members are able to promote metastatic colonization, which also demonstrates miR‐200 family promote the MET in the premetastatic niche.^177^ Besides, recent research indicates that the miR‐203a‐3p derived from hepatocyte exosomes can lead to the E‐cadherin re‐expression of colorectal cancer cells via inhibiting Src expression, resulting in MET process and finally liver metastases formation.^178^

### 5.2 Intracellular molecular mechanisms influencing EMP

#### 5.2.1 Metabolic reprogramming

To adapt to increasing nutrient needs to gain the mesenchymal state from epithelial state in the tumor microenvironment with poor nutrition, metabolic reprogramming including changes in glucose, fatty acid and amino acid metabolism is needed for tumor cells in the primary niche.^179^, ^180^ Besides, the metabolic reprogramming in tumor cells undergoing EMT can facilitate their chemotherapy resistance.^181^ Compared with normal cells, tumor cells show increased glucose uptake and aerobic glycolysis instead of OXPHOS to meet their biosynthesis demand, called Warburg effect.^182^, ^183^ In the initial step of the glycolysis, the enhanced retromer‐controlled translocation of glucose transporter type 1 (GLUT1), a glucose transporter that exhibits the ability to facilitate glycolysis and cancer progression, to the PM is essential for glucose uptake.^184^ Overexpression of GLUT1 has been shown to enhance the expression of MMP2 and participate in the process that TGF‐β1 induces EMT of tumor cells.^185^ Lactate dehydrogenase A (LDHA) is the key enzyme that can convert pyruvate into lactate during glycolysis, which involves the last step of glycolysis.^186^, ^187^ The elevated LDHA can promote EMT of tumor cells through upregulating the expression of ZEB2 and activating of TGF‐β due to the low extracellular pH induced by the secretion of lactate.^188^, ^189^

#### 5.2.2 Domesticated autophagy and anoikis

Autophagy, a highly conserved cellular homeostasis mechanism that facilitates degradation of damaged cellular constituents and nutrient recycling, occurs in almost every cell and is increased under conditions of cellular stress from inside or outside the cell, such as nutritional starvation, hypoxia, and oxidative stress.^190^ Dysregulation of autophagy has been observed to be a highly relevant factor for various diseases especially cancer, where the function of autophagy depends on the different stages during tumor progression.^191^ However, autophagy can also be domesticated by tumor cells as a significant self‐protective mechanism to meet the tumors benefits with evolved phenotypic plasticity to survive under stress conditions in the setting of advanced cancer.^192^ Intriguingly, the action of EMT‐mediated signaling pathways on autophagy may collectively provide an interplay panorama between these two processes during tumor deterioration. In pancreatic cancer stem‐like cells, autophagy activated by HIF‐1α promotes EMT and metastasis ability via downregulating E‐cadherin as well as upregulating Vimentin and MMP‐9 levels.^193^ Besides, autophagy is also found to promote EMT by TGF‐β1/SMAD3 signaling pathway, while TGF‐β1 can also activate autophagy in turn, resulting in the formation of a positive feedback loop to synergistically enhance EMT process as well as the migration of tumor cells.^194^ Moreover, other EMT‐related signaling pathways can also activate autophagy such as Wnt/β‐catenin, Notch, and HIF‐1α.^195^

Expression of autophagy associated gene Beclin‐1 has been reported to be correlated to the increasing populations of CTCs and autophagy is responsible for the anoikis resistance.^166^, ^196^, ^197^ Anoikis is a type of apoptotic cell death that can be triggered by ECM detachment.^198^ After initiating EMT in the primary niche, tumor cells intravasate into vascular system through remodeled ECM and become CTCs, most of which are found to be in the p‐EMT state.^165^, ^166^ The mesenchymal properties of p‐EMT state tumor cells with epithelial adhesion molecules allow them to migrate through ECM with enhanced cell–cell adhesion, which may contribute to collective migration.^17^ The collective migration of CTC clusters is significantly more effective than single‐cell migration and thought to be the key mediator of tumor metastasis.^199^ Compared with single CTC, CTC clusters with p‐EMT state are resistant to anoikis.^199^, ^200^ Thus, how autophagy participates in the resistance of anoikis and formation of CTCs–platelet complexes in the vascular niche to maintain the survival of CTC clusters will be discussed as follows.

The interaction between tumor cells depends on the focal adhesion (FA) complexes, PM‐associated complexes, which mediate the physical connection between the cell cytoskeleton and ECM via adhesion proteins.^201^ Focal adhesion kinase (FAK) and Src, both of which belong to cytosolic nonreceptor tyrosine kinases, can regulate the FA complexes and significant signaling pathways for survival and metastasis of tumor cells.^202^, ^203^, ^204^ When detaching from the ECM, apoptotic signaling pathways in tumor cells are activated, leading to anoikis.^205^ During the detachment of CTCs from the ECM, Src is inactive and enhances the activation of adenosine monophosphate‐activated protein kinase to trigger autophagy to support the survival of CTCs.^206^ Besides, the disrupted Src/FAK pathway has been found to lead to apoptosis of tumor cells, while autophagic degradation of active Src avoids the apoptosis.^207^ In addition, autophagy can be activated by the protein kinase R‐like endoplasmic reticulum kinase (PERK) induced by oxidative stress during ECM detachment. PERK upregulates the expression of autophagy‐related gene genes to induce autophagy through eukaryotic translation initiation factor 2α (eIF2α)‐activating TF 4‐C/EBP homologous protein (CHOP) pathway, and the activated autophagy facilitates tumor cells to better survive under oxidative stress as well as resist anoikis.^208^

In conclusion, various intracellular and extracellular molecular mechanisms are crucial for the EMT, p‐EMT, and MET of tumor cells, all of which are utilized by tumor cells to adapt to the dynamic surrounding niches during tumor deterioration. Targeting these molecular mechanisms may lead to developing novel therapeutic approaches to regulate EMP.

## 6 EMP AND THERAPEUTIC RESISTANCE

Therapeutic resistance is widely recognized as a crucial contributor to therapeutic failure. During the treatments with traditional chemotherapy and novel targeted therapy as well as immunotherapy on cancers, various processes such as EMT involve the development of cancer cells resistance to these therapies. The EMP‐induced phenotypic heterogeneity of tumor is tightly associated with therapeutic resistance.^209^ Recent research using RNA sequence analysis indicates that therapy sensitive cells exhibit MET, while therapy resistant cells exhibit EMT.^13^ Increasing evidence suggests that both EMT‐TFs such as Snail and Twist as well as EMT‐related signaling pathways including TGF‐β, Wnt, and Notch are correlated with the therapeutic resistance.^5^, ^210^, ^211^ Here, we summarize the EMP‐induced resistance to chemotherapy and targeted therapy as well as the implications of EMP in immune evasion and immunotherapy resistance.

### 6.1 EMP‐associated resistance to chemotherapy and targeted therapy

Chemotherapy such as paclitaxel or cisplatin and targeted therapies such as targeted kinase inhibitors are common therapies for the treatment of solid tumor. The changes of EMP biomarkers and signaling pathways regulating EMP have been observed to participate in resistance to chemotherapy and targeted therapies in various cancers. Cisplatin is the representative of platinum‐based drugs that are widely used in various cancers including ovarian cancer, bladder cancer, and colorectal adenocarcinoma. The cisplatin‐resistant colorectal adenocarcinoma cells are found to exhibit mesenchymal features such as increased N‐cadherin and reduced E‐cadherin. Interestingly, these mesenchymal features are mediated by the cisplatin‐induced TGF‐β signaling, which in turn contributes to the cisplatin resistance.^212^ Paclitaxel is one of the most frequently employed chemotherapeutic agents in the clinic treatment of breast cancer and lung cancer. However, its effects are limited by paclitaxel resistance correlated with EMT. AlkB homolog 5 (ALKBH5), a N6‐methyladenosine demethylase that can repress tumor progression, is downregulated in paclitaxel‐resistant non‐small cell lung cancer. The downregulated ALKBH5 is responsible for the cell migration inducing hyaluronidase 1‐mediated EMT, which leads to the acquisition of paclitaxel resistance.^213^ TNF‐related apoptosis‐inducing ligand (TRAIL) is a chemotherapeutic agent that can specifically induce the apoptosis of cancer cells without killing effects on normal cells.^214^ The inhibition of epithelial marker E‐cadherin contributes to the emergence of TRAIL resistance, while E‐cadherin re‐expression can reverse EMT and promote the sensitivity of cancer cells to TRAIL via facilitating TRAIL apoptotic pathway.^215^ Besides chemotherapeutic resistance, EMT also contributes to the resistance to targeted therapies. Targeting molecules of mitogen‐activated protein kinase (MAPK) pathway with MAPK inhibitors is a promising therapy for the treatment of metastatic melanoma. ZEB1 overexpression is found to mediate tumor resistance to MAPK inhibitors via driving the phenotype switch of melanoma cells.^216^ Sorafenib is a tyrosine kinase inhibitor crucial for the improved overall survival of advanced hepatocellular carcinoma patients. Recent research indicates that SMYD3 overexpression can contribute to the EMT‐related gene overexpression in sorafenib‐resistant hepatocellular carcinoma cells. The upregulated SMYD3 is found to promote the expression of ZEB1, Snail, and MMP9 to induce EMT via interacting with SMAD2/3, resulting in the sorafenib resistance of hepatocellular carcinoma.^217^

### 6.2 EMP in immune evasion and resistance to immunotherapy

In recent years, immunotherapy has appeared as a novel approach that develops rapidly, which aims to restore or facilitate the host immune response to treat cancer. Immunotherapy, including immune checkpoint inhibitors (ICIs) and chimeric antigen receptor (CAR)‐T cells therapy, has contributed to long‐term effects and prolonged patient survival in various types of cancer.^218^ However, the immunotherapy response rates are relatively lower in some cancer patients due to the immune evasion and immunotherapeutic resistance modulated by EMP. Here we summarize the EMP‐related immune evasion induced by immunosuppressive microenvironment as well as escape of immune surveillance and immunotherapy resistance.

The immunosuppressive microenvironment commonly comprises the aberrant landscape of immune cells infiltrates and increased immunosuppressive molecules. Among immune cells, natural killer (NK) cells and cytotoxic CD8^+^ T cells exhibit detection and killing functions, while regulatory T cells (Tregs) and TAMs exhibit immunosuppressive functions. In non‐small cell lung cancer, EMT is found to enhance the infiltration of Tregs and reduction of CD8^+^ T cells.^219^ Recent research shows that the EMT‐TF ZEB1 can facilitate secretory vesicle trafficking via activating exocytotic Rabs to promote the autotaxin‐mediated exhaustion of CD8^+^ T cells in lung cancer, resulting in immunosuppression in the tumor microenvironment.^220^ CD73 is an ectonucleotidase often expressed in mesenchymal cancer cells and a potential target for immunotherapy since it can participate in the generation of the immunosuppressive molecule adenosine.^221^, ^222^ Snail is reported to upregulate the expression of adenosine, which is responsible for the inhibited antitumor function of CD8^+^ T and NK cells as well as the promoted immunosuppressive function of Tregs and TAMs.^222^ Programmed death‐ligand 1 (PD‐L1), one of the most frequently upregulated checkpoint proteins in tumor, can bind to programmed death receptor 1 (PD‐1) on CD8^+^ T cells, which leads to the inhibition of the survival and effector functions of CD8^+^ T cells.^223^ The increased expression is associated with inflammatory factors such as interferon‐gamma (IFN‐γ). The IFN‐γ‐induced PD‐L1 is considered to promote EMT and impair MET, whereas EMT may in turn impact the expression of PD‐L1.^224^ In addition, the p‐EMT state CTC clusters can interact with activated platelets in the vascular system, contributing to forming microthrombi (CTCs–platelet complex) that can protect CTCs from immune surveillance.^225^ The platelets attached on the surface of CTCs form the physical shield to protect CTCs from the immune attack from NK cells during circulation in the blood vessels.^226^ Besides, the major histocompatibility complex (MHC)‐I molecules of platelets are transferred to the surface of CTCs to escape the immune surveillance, since NK cells recognize and kill the target cells with less or without expression of MHC‐I molecules.^227^

Besides immune evasion, EMP is also involved in the resistance to ICIs including PD‐1, PD‐L1, and CTLA‐4 inhibitors as well as CAR T cells therapy. EMT status is tightly related to the overexpression of PD‐1, PD‐L1, and CTLA‐4 in cancer cells, all of which can inhibit the effector functions of CD8^+^ T cells.^219^, ^228^ Recent research shows tumor exhibiting mesenchymal‐like is T cell‐excluded with CTLA4 blockade resistance compared with epithelial‐like tumor, indicating EMT is a significant player in the resistance to immunotherapy.^229^ Gli2 is a TF of the EMT‐related Hedgehog signaling pathway that can impact on the response to anti‐PD‐1 immunotherapy in tumor. The Gli2 activation in tumor exhibiting EMT is found to promote the resistance to PD‐1 inhibitors via upregulating Wnt signaling.^230^ Interestingly, EMT can contribute to the occurrence of promoted immune‐evading phenotype resistant to PD‐1 antibody along with autophagy.^231^, ^232^ The inhibition of YAP1, a downstream of EMT‐related Hippo signaling pathway, can facilitate the efficacy of anti‐PD‐1 immunotherapy via decreasing the autophagic flux in hepatocellular carcinoma.^233^ Besides, autophagic degradation of PD‐L1 can contribute to the repression of the immune escape in non‐small‐cell lung cancer, suggesting that the role of autophagy in EMT/checkpoint proteins axis is complicated.^234^ B7‐H4, a type 1 transmembrane protein that exerts crucial effects on the repression of immune response mediated by T cell, is found to be dynamically downregulated in the advancement of breast cancer. The dynamic downregulation of B7‐H4 promotes EMT in breast cancer cells and simultaneously enables breast cancer cells to escape from the cytotoxicity of B7‐H4 CAR‐T cells.^235^

In conclusion, EMP is deeply involved in the therapeutic resistance as well as immune evasion of tumor, both of which are the key factors drive tumor progression. Therefore, therapeutic targeting EMP is a promising approach to overcome the treatment failure in cancer and has substantial clinical significance.

## 7 THERAPEUTIC STRATEGIES TARGETING EMP

As discussed in previous chapters, EMP impacts greatly on multiple aspects of tumor deterioration including metastasis, therapeutic resistance, and immune evasion. With the increasing understanding of mechanisms that modulate EMP, researchers have developed a range of therapeutic approaches to target EMP, overcoming the EMP‐driven tumor deterioration. Here, we will concentrate on EMP‐targeted therapeutic approaches based on the EMP‐related mechanisms mentioned above, including small molecule inhibitors, immunotherapeutic approaches, and combination therapies.

### 7.1 Small molecule inhibitors targeting EMT‐inducing factors

With key molecules in the EMT‐TFs and EMT‐related signaling pathways as intervention targets, various small molecule inhibitors have been developed to regulate EMP in cancers. Omeprazole is a proton pump inhibitor found to exhibit anticancer activity and ability to significantly suppress the invasive as well as migratory properties in aggressive cancer cells. Omeprazole is reported to bind to the Snail protein physically and inhibit Snail‐driven EMT via disrupting CBP/p300‐mediated Snail acetylation and promoting ubiquitin–proteasome pathway‐mediated Snail degradation, resulting in decreased tumor invasion and metastasis.^236^ 2‐(3‐Hydroxyphenyl)‐5‐methylnaphthyridin‐4‐one (CSC‐3436) is a newly synthesized flavonoid derivative that exhibits effective cytotoxicity against non‐small‐cell lung cancer cells and triple‐negative breast cancer cells. In head and neck squamous cell carcinoma cells, CSC‐3436 is reported to inhibit the Twist expression to downregulate the expression of B cell‐specific Moloney murine leukemia virus integration site 1 (Bmi1), a biomarker of tumor‐initiated cells. The inhibited Twist/Bmi1 axis leads to the downregulation of Akt/β‐catenin signaling, thereby inhibiting the Twist‐mediated EMT process.^237^ Decitabine, a DNMT inhibitor, is shown to reverse TGF‐β1‐mediated EMT of non‐small‐cell lung cancer cells via regulating the miR‐200/ZEB axis.^238^ Ursolic acid is a known triterpenoid compound that occurs naturally and exhibits anticancer activities against various types of cancer cell. Recent research indicates that ursolic acid can inhibit the TGF‐β‐induced EMT in glioblastoma.^239^ Besides, targeting the EGFR involved in EMT with the EGFR inhibitor gefitinib can inhibit the tamoxifen resistance, invasion and migration via downregulating Snail and Twist in breast cancer.^240^ In addition, treatment with the novel and effective PI3K inhibitor serabelisib is reported to suppress the EMT process via PI3K/Akt/E‐cadherin signaling pathway, thereby inhibiting the migration of hepatoma cells.^241^

Besides EMT‐TFs and EMT‐related signaling pathways, targeting the EMT‐associated molecular mechanisms can also regulate the EMP of tumor. 6‐Gingerol is a major component of ginger that exhibits antitumor activity. Recent research indicates that 6‐Gingerol can block the nuclear accumulation of HIF‐1α in hypoxic conditions via modulating the interaction of HIF‐1α with heat shock protein 90 (HSP90), a molecular chaperone that stabilizes HIF‐1α, leading to the inhibition of EMT.^242^ Notopterol, a bioactive compound that belongs to furanocoumarin, is found to inhibit the proinflammatory cytokine IL‐17‐induced EMT to reduce the invasion of lung cancer cells.^243^ Pyruvate kinase2 (PKM2) is a rate‐limiting enzyme of glycolysis that can promote glycolysis and lead to metastasis.^244^ A recent study shows that the fungal metabolite cerulenin can target PKM2 via inhibiting EGFR 2 to downregulate the EMT in breast cancer cells.^245^ FRAX486, a type I p21‐activated kinase (PAK) inhibitor, is identified as a potent candidate inhibitor of autophagy/EMT after screening over 2000 small molecule chemicals for dual autophagy/EMT inhibitors in a recent study. It is reported that FRAX486 can inhibit the PAK2‐mediated autophagosome–lysosome fusion of autophagy to block the autophagic degradation of E‐cadherin, resulting in inhibition of EMT and metastasis of triple‐negative breast cancer cells.^246^ These small molecule inhibitors are summarized in Table 1.

**TABLE 1:** Current small molecule inhibitors targeting EMT‐inducing factors.

Inhibitors	Targets or pathways	Cancer types	Effects	Animal experiments	References
Omeprazole	Snail	Colon cancer	Suppressing growth and metastasis	Mouse xenograft model and metastasis model	^236^
CSC‐3436	Twist/Bmi1	Head and neck squamous cell carcinoma	Inhibiting stemness, invasion, and migration	Mouse tail vein metastasis assay	^237^
Decitabine	miR‐200/ZEB	Non‐small‐cell lung cancer	Reducing metastasis	Mouse xenograft model	^238^
Ursolic acid	TGF‐β	Glioblastoma	Inhibiting growth	Mouse xenograft model	^239^
Gefitinib	EGFR	Breast cancer	Inhibiting tamoxifen resistance, migration, and invasion	Not applicable	^240^
Serabelisib	PI3K/Akt/E‐cadherin	Hepatoma	Inhibiting migration	Not applicable	^241^
6‐Gingerol	HIF‐1α	Lung cancer	Inhibiting growth and metastasis	Mouse xenograft model	^242^
Notopterol	IL‐17	Lung cancer	Reducing proliferation and invasion	Not applicable	^243^
Cerulenin	PKM2	Breast cancer	Inhibiting proliferation, migration, invasion, and glycolysis	Not applicable	^245^
FRAX486	PAK2	Triple‐negative breast cancer	Inhibiting metastasis	Mouse tail vein metastasis assay	^246^

Abbreviations: EGFR, epidermal growth factor receptor; IL‐17, interleukin‐17; PAK2, p21‐activated kinase2; PI3K, phosphatidylinositol‐3‐kinase; PKM2, pyruvate kinase2.

### 7.2 Immunotherapeutic approaches to modulate EMP for enhanced antitumor immunity

In addition to small molecule inhibitors, immunotherapeutic approaches that modulate the EMP of tumor have also been studied recently. A recent study suggests that the anti‐PD‐L1 monoclonal antibody treatment can downregulate the EMT‐TF ZEB1 expression to inhibit EMT.^247^ Dovitinib, an inhibitor of multiple receptor tyrosine kinases, is reported to induce the activation of tumor‐intrinsic SNAI1/2‐IFN‐γ signaling and suppress EMT in another recent study, which promotes T cell recruitment and makes mesenchymal‐like tumor to be sensitive to CTLA4 blockade.^229^ *Plasmodium* immunotherapy is a novel form of immunotherapy that has antitumor effects. Recent research indicates that treatment with *Plasmodium chabaudi* ASS infection and gemcitabine can block the CXCR2/TGF‐β‐mediated PI3K/Akt/GSK‐3β signaling pathway to inhibit EMT process of tumor cells.^248^

### 7.3 Combination therapies to overcome EMP‐mediated therapeutic resistance

Due to the tumor resistance to monotherapy, it is important to consider combination therapies that have been developed by researchers to overcome the therapeutic resistance mediated by EMP. Evodiamine is a natural alkaloid component that exhibits powerful antitumor activity. It is reported that combinatory treatment of cisplatin and evodiamine can inhibit EMT via downregulating β‐catenin expression, resulting in overcoming the cisplatin resistance of non‐small‐cell lung cancer cells.^249^ AXL is a receptor tyrosine kinase crucial for the maintenance of mesenchymal phenotype in various cancers. The AXL inhibitor TP‐0903 can induce MET and synergize with artesunate to promote the cytotoxicity of artesunate in triple‐negative breast cancer cells.^250^ Besides, the combination of the PI3K inhibitor alpelisib and eribulin can downregulate the PI3K/Akt pathway and inhibit EMT, leading to highly effective inhibition on paclitaxel‐resistant endometrial cancer cells.^251^ Moreover, a newly developed anti‐TGF‐β/vascular endothelial growth factor (VEGF) bispecific antibody Y332D that exhibits reversing EMT activity can be combined with anti‐PD‐1 therapy to enhance the T cells infiltration and facilitate the T cells cytotoxicity in murine tumor model.^252^ In summary, a number of small molecule inhibitors, immune‐related therapeutic approaches, and combination therapies targeting EMP have been reported in recent years. Besides, we summarized clinical trials associated with EMP in Table 2. However, it is necessary for further investigations to comprehensively understand the EMP pathogenesis and develop more precise and effective therapeutics targeting EMP.

**TABLE 2:** Clinical trials associated with EMP.

NCT	Drug	Phase	Cancer type	Intervene strategies
NCT05550415	Simvastatin	II	Triple‐negative breast cancer	Inhibiting EMT process
NCT01861054	Reparixin	II	Breast cancer	Inhibiting EMT process
NCT02001974	Paclitaxel+reparixin	I	Metastatic breast cancer	Inhibiting EMT process
NCT01952054	Denosumab	II	Breast cancer	Inhibiting EMT process
NCT02602938	Aspirin	II	Advanced breast and colorectal cancer	Inhibiting EMT process
NCT02913859	Hormonal therapy	II	Prostate cancer	Inhibiting EMT process
NCT02412462	AB‐16B5	I	Advanced solid malignancy	Inhibiting EMT process
NCT06203821	NP137	I	Pancreatic cancer	Inhibiting EMT process
NCT05445791	Metformin hydrochloride	III	Non‐small cell lung cancer	Inhibiting EMT‐induced drug resistance
NCT06225843	AB‐16B5+FOLFOX	II	Metastatic colorectal cancer	Inhibiting EMT process
NCT04364620	AB‐16B5+docetaxel	II	Non‐small cell lung cancer	Inhibiting EMT process
NCT04664829	Bexarotene+capecitabine	I	Triple‐negative breast cancer	Reversing EMT process
NCT02424617	Erlotinib+bemcentinib	I/II	Non‐small cell lung cancer	Killing cells undergoing EMT
NCT01534585	Icotinib+intensity‐modulated radiotherapy	I/II	Nasopharyngeal carcinoma	Inhibiting EMT process
NCT05242965	CD105/Yb‐1/SOX2/CDH3/MDM2‐polyepitope plasmid DNA Vaccine	II	Non‐small cell lung cancer	Killing cells undergoing EMT

Abbreviations: EMT, epithelial–mesenchymal transition; FOLFOX, a chemotherapy regimen including folinic acid, fluorouracil, and oxaliplatin; MDM2, mouse double minute 2 homolog; NCT, number of clinical trial; SOX2, sex determining region Y‐box 2.

## 8 FUTURE PERSPECTIVES AND EMERGING TECHNOLOGIES

Despite extensive efforts to study the signatures of EMP and mechanisms driving EMP, some aspects of EMP remain poorly understood such as the EMP‐induced heterogeneity in the tumor microenvironment and how this heterogeneity progresses at different stages of tumor. Recently, with the fast development of sing‐cell sequencing and spatial transcriptomics, these technologies are being more frequently used by researchers to study the EMP‐induced heterogeneity. Single‐cell RNA sequencing (scRNA‐seq) is thought to be crucial for the assessment of EMT in tumors since bulk RNA signature is not able to describe the heterogeneous tumor cell subpopulations in detail.^253^ A recent study developed a scRNA‐seq data‐driven computational framework named COMET (Cell line‐specific Optimization Method of EMT Trajectories) to predict EMT‐trajectories. COMET can be utilized to infer the dynamic phenotypic transitions induced by EMP, including EMT, p‐EMT, and MET, to predict the timing and distribution of phenotypic heterogeneity within tumors.^254^ Besides, spatial transcriptomics can also analyze RNA levels in a spatial context to reveal tissue heterogeneity.^255^ Another recent study employed spatial transcriptomics and single cell datasets to investigate the spatial heterogeneity of EMT and analyzed the various interactions between EMT and NK cells as well as fibroblasts in the tumor microenvironment.^256^ Moreover, it is found that the EMT in different stages of tumor is associated with the various spatiotemporal distributions of the immune microenvironment by using scRNA‐seq and spatial transcriptomics.^257^

With the increasing understanding of EMP complexity and restriction of traditional therapies, researchers have developed various novel therapeutic interventions targeting EMP such as nanodrugs and RNA‐based therapy, both of which exhibit great potential to improve therapy against cancer. In recent years, rapid progress has been achieved in nanotechnology, which can be employed for targeted drug delivery. Nanoparticle delivery with enhanced capacity to kill tumor also exhibits excellent biocompatibility and high stability.^258^ Macrophage membrane‐coated nanoparticles (MϕNP) is proposed to subvert the immunosuppressive function of TAMs via inhibiting immunosuppressive cytokines recently. It is reported that a newly developed MϕNP loaded with SD‐208, a TGF‐βR1 kinase inhibitor, can selectively target TAMs and cancer cells to simultaneously reverse the immunosuppressive tumor microenvironment and EMT, exhibiting robust anticancer effect in combination with anti‐PD‐1 antibodies.^259^ Besides, our previous study designed and synthesized a hyaluronic acid‐modified liposomes loaded with cisplatin and hesperetin to inhibit EMT and metastasis of triple‐negative breast cancer via downregulating PI3K/Akt/mTOR signaling.^260^ RNA‐based therapeutics such as siRNA and miRNA have been employed to regulate the gene expression of tumor cells. However, due to the early degradation in vivo environment, RNAs depend on efficient delivery system to achieve antitumor effects.^261^ A recent study designed a hyaluronidase and glutathione dual‐bioresponsive nanosystem to deliver TGF‐β siRNA effectively, which can inhibit the EMT and remodel the tumor immune microenvironment.^262^ Exosomes, which are naturally secreted by the body's cells, are also promising nanocarriers for RNA‐based therapy.^261^ For instance, exosomes isolated from adipose‐derived MSCs can deliver miR‐381 mimic to triple‐negative breast cancer cells, which significantly inhibit the expression of EMT‐related genes and proteins, suppressing the aggressiveness of triple‐negative breast cancer.^263^

## 9 CONCLUSION

EMP is a key signature of malignant tumor that drives tumor cells to switch their cellular phenotype between epithelial and mesenchymal phenotype, including EMT, p‐EMT, and MET, to adapt to various stress conditions. These transitions contribute to the morphological heterogeneity and functional heterogeneity of tumor cells, both of which are tightly associated with the complicated progress of tumor. EMP is found to be regulated by various EMT‐TFs such as Snail, ZEB, and Twist as well as a number of EMT‐related signaling pathways including TGF‐β, Wnt/β‐catenin, and Notch. Interestingly, several other intracellular and extracellular molecular mechanisms in the dynamic tumor microenvironment such as hypoxia, inflammation, remodeled ECM, stromal cells, EVs, metabolic reprogramming, domesticated autophagy, and anoikis are also found to drive EMP. These suggest that EMP is an extremely complex mechanism, and more efforts are needed to study the interaction of various factors that regulate EMP for better understanding of the occurrence and development of tumor EMP.

The EMT, p‐EMT, and MET induced by various EMP‐related factors have been demonstrated to deeply participate in the deterioration of tumor, including metastasis, therapeutic resistance and immune evasion. To overcome the EMP‐driven tumor deterioration, researchers have developed a range of small molecule inhibitors, immune‐related therapeutic approaches, and combination therapies to target the key biomarkers or crucial signaling pathways of EMP during tumor progression. However, due to the complicated intratumoral heterogeneity induced by EMP, more precise and effective therapies remain to be developed in the future. Besides, it is also significant to consider how to achieve personalized clinical treatments and improve medication compliance.

With the rapid development of transcriptomic profiling technique, sing‐cell sequencing and spatial transcriptomics are used to study the EMP heterogeneity. Recent studies have utilized these two technologies to predict the timing and distribution of phenotypic heterogeneity within tumors and analyze the interaction between EMT and the tumor microenvironment. Also, it is noteworthy that the EMT states are multidimensional and nonlinear, and thus, how to rigorously quantify the EMT states of tumor cells need to be studied in the future. The advance in experiment technologies such as more sensitive lineage tracing techniques and new models may boost the understanding of EMP. In conclusion, analyzing the right EMT state in different stages and finely regulating the EMP during the dynamic progression of tumor via developing novel EMT‐target drugs or employing suitable delivery system in the future may be the key step toward conquering tumor and provide precise therapeutic strategy for cancer.

## AUTHOR CONTRIBUTIONS

Xiangpeng Wang and Yuanyan Liu wrote and conceived the manuscript. Xiaoxia Xue, Mingshi Pang, Liuchunyang Yu, Jinxiu Qian, Xiaoyu Li, and Meng Tian searched and collected literature. Yuanyan Liu, Aiping Lyu, Cheng Lu, and Xiangpeng Wang revised the manuscript. All authors read and approved the final manuscript.

## CONFLICT OF INTEREST STATEMENT

The authors declare that there are no potential conflict of interest.

## ETHICS STATEMENT

Not applicable.

## Data Availability

Not applicable.
